# Risk Factors for Positive Resection Margins in Breast-Conserving Surgery for Breast Cancer—Retrospective Analysis

**DOI:** 10.3390/cancers16172930

**Published:** 2024-08-23

**Authors:** Rares Georgescu, Flavian Tutuianu, Orsolya Bauer, Anca Toganel, Zalan Benedek, Eugeniu Darii, Sabin Turdean, Cristina Tutuianu Radoi

**Affiliations:** 1 Department of Surgery, University of Medicine, Pharmacy, Science and Technology “G.E. Palade” Targu Mures, 540139 Targu Mures, Romania; 2 Department of Gynecology, University of Medicine, Pharmacy, Science and Technology “G.E. Palade” Targu Mures, 540139 Targu Mures, Romania; 3 Department of Oncology, University of Medicine, Pharmacy, Science and Technology “G.E. Palade” Targu Mures, 540139 Targu Mures, Romania; 4 Department of General Surgery, Oncocard Brasov, 500052 Brasov, Romania; 5 Department of Pathology, University of Medicine, Pharmacy, Science and Technology “G.E. Palade” Targu Mures, 540139 Targu Mures, Romania; sabin.turdean@umfst.ro

**Keywords:** early-stage breast cancer, resection margins, positive margins

## Abstract

**Simple Summary:**

This research aims to identify preoperative factors that may predict positive resection margins in patients undergoing breast-conserving surgery for early-stage breast cancer. By retrospectively analyzing patient records from 2009 to 2017, the study investigates preoperative factors associated with positive resection margins. The findings indicate that neoadjuvant chemotherapy and the presence of ductal carcinoma in situ are significantly associated with positive resection margins. These results underscore the importance of these preoperative factors in surgical planning and management, potentially leading to improved outcomes for breast cancer patients.

**Abstract:**

The primary objective of this study was to identify preoperative factors that could be associated with positive resection margins. We also tried to analyze the local recurrence and overall survival in patients who received conservative treatment for early-stage breast cancer and correlate these parameters with preoperative factors. A retrospective examination was conducted on the medical records and pathological reports of 143 patients who underwent breast-conserving surgery (BCS) for breast cancer in our department from 2009 to 2017. Postoperative outcomes were assessed through phone contact and statistical analyses, including GraphPad Prism, and Fisher’s exact test, the Chi-square test, and the log-rank test were employed. The results revealed positive resection margins in 7.69% (11 cases) of the 143 patients, with an overall mortality rate of 16.66% for those with positive margins and 6.59% for those with negative margins. Statistical analysis indicated no significant differences in the overall (*p* = 0.5) or specific (*p* = 0.53) survival between the positive and negative margin groups. The positive margins were significantly associated with neoadjuvant chemotherapy (*p* < 0.0001) and the presence of ductal carcinoma in situ (DCIS) (*p* = 0.01). Among the analyzed factors, two out of sixteen were significantly linked to positive resection margins in BCS, emphasizing their importance in surgical management planning for early-stage breast cancer.

## 1. Introduction

Breast cancer represents 25% of cancers diagnosed in women all over the world, with significant differences in incidence and mortality across countries and continents and 12% of all cancers as a frequency. However, mortality has decreased considerably due to screening programs and oncological treatments [1].

Breast-conserving therapy is the gold standard treatment for early-stage breast cancer. The most important risk factors for local recurrence after breast-conserving surgery are the positive resection margins and the young age of the patient [2,3].

The impact of margin status on local recurrence was analyzed in subgroups stratified by age and hormone receptor status, which were identified as independent risk factors for local recurrence [4].

However, the local recurrence rate was lower than in the past, about 0.5% per year [5,6]. Positive margins were associated with higher local recurrence with marginal significance in all the patients. In the patients aged <60 years, positive margins were associated with poor local control, despite a higher dose of >65 Gy EQD2. In contrast, no significant effect of positive margins on the local control was observed in patients aged ≥60 years [4].

Patients with a tumor on ink margins had a pooled overall distant recurrence risk of 25.4% and a local recurrence risk of 15.9%, whereas patients with a tumor at or close to inked margins had a distant recurrence risk of 8.4% and a local recurrence risk of 8.8%. Patients with negative margins had a distant recurrence of 7.4% and a local recurrence risk of 3.9% [7].

There is no consensus regarding oncologically appropriate surgical resection margins. According to the SSO-ASTRO Margin Guideline by Monica Morrow, the principle of “no ink on tumor” for invasive breast tumors is accepted [8].

Changes in the understanding of breast cancer biology and the widespread use of adjuvant systemic therapy for early breast cancer have also affected attitudes toward margins [9].

The National Surgical Adjuvant Breast and Bowel Project B-24 trial showed that the adjuvant tamoxifen reduced ipsilateral breast tumor recurrence rates in patients with positive margins to levels similar to those in patients with negative margins [10].

The aim of this study was to identify preoperative factors that could be associated with positive resection margins. Also, we analyzed the overall survival in patients who received conservative treatment for early-stage breast cancer, and we analyzed if re-excised positive margins are associated with local recurrence compared to patients with only one surgery.

## 2. Materials and Methods

This is a retrospective study performed in the Surgical Clinic of Mures County Clinical Hospital. We used data from patients who were diagnosed with breast cancer and benefited from conservative breast treatment between 2009 and 2017. All the patients included in the study were operated on by the same surgeon and benefited from postoperative radiotherapy after BCS.

We used the clinic’s database, the surgical notes, and the histopathological reports to collect all the information.

The status of the surgical resection margins was established according to the recommendation “no ink on tumor”.

Regarding the postoperative outcome, we evaluated the local recurrence and the overall survival by phone contact with the patients following ultrasound and mammography investigations, which were carried out periodically. The follow-up period was between 20 and 120 months. Out of a total of 143 cases, 46 patients could not be reached; therefore, 97 patients were completely included in the study, and 143 were included only in determining the preoperative parameters associated with the positive resection margins.

Regarding the surgical intervention of the axilla, the number of identified or positive axillary lymph nodes was not quantified because they did not represent the purpose of this study. All the patients had mammography before and after oncological treatment or before surgery if it was performed primarily. Also, all patients underwent breast ultrasound before surgery.

Regarding neoadjuvant oncological treatment, we do not have data on the type of chemotherapy and the number of cycles performed. All the patients included in the study followed the complete neoadjuvant treatment protocol.

Regarding the surgical intervention of the axilla, the number of identified or positive axillary lymph nodes was not quantified because they did not represent the purpose of this study. All the patients underwent sentinel lymph node biopsy or axillary lymphadenectomy depending on the preoperative clinical and ultrasound status of the axilla. We did not use frozen sections.

Statistical data analysis was performed using GraphPad Prism 6 using Fisher’s exact test, the Chi-square test, and Kaplan Meier survival curves. The results were interpreted as statistically significant when *p* < 0.05.

## 3. Results

Of the 143 patients included in this study, in 11 patients, positive resection margins were identified after wide excision (7.6%); the remaining 132 patients had negative margins.

When comparing the two groups of patients, no statistically significant differences were observed in terms of the overall survival and the specific survival.

Of the total patients followed, the overall mortality was 16.6% for patients with positive resection margins and 6.5% for patients with negative resection margins.

**Figure 1:**
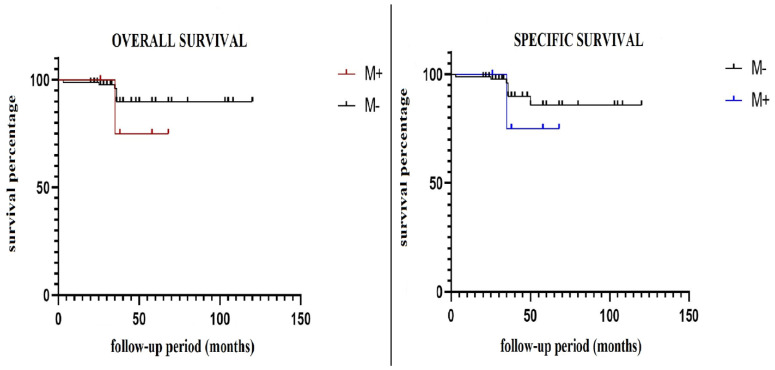
Overall and Specific Survival.

For the overall survival, the value of *p* was 0.50, and for the specific survival, *p* = 0.53, which was statistically insignificant (Figure 1).

In total, 6 of the 11 patients with positive resection margins benefited from a second wide excision, while 3 patients underwent mastectomy because of the impossibility of obtaining adequate resection margins. A total of 5 of the 97 patients questioned developed metastases. Local recurrence was not confirmed in any patients during the follow-up period.

We retrospectively analyzed 16 preoperative factors, of which 2 were statistically significantly associated with the positive resection margins.

**Figure 2:**
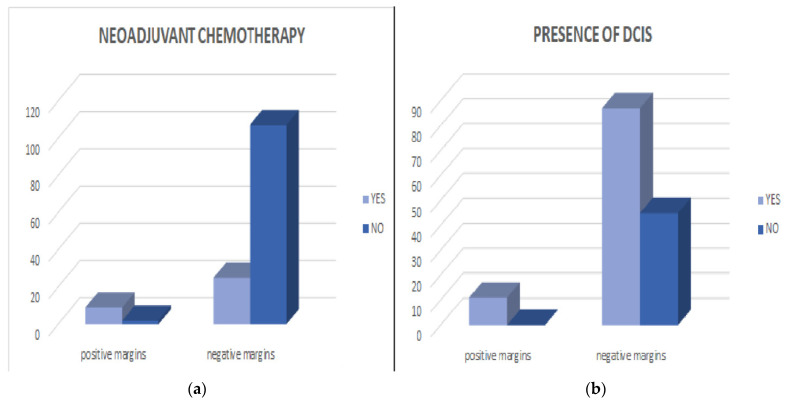
Neoadjuvant chemotherapy and Presence of DCIS

Of the 143 patients, according to the protocols and following a discussion with the oncological tumor board, 34 patients required neoadjuvant chemotherapy; the remaining 109 did not, so they benefited from surgery in the first place. In total, 34 patients followed neoadjuvant chemotherapy, of which 9 had positive resection margins after the wide excision, which represents 26.4% compared to 1.8% of positive resection margins in patients who underwent primary surgery. The risk of a patient having positive resection margins in breast-conservatory surgery is almost 20 times higher for those who have undergone neoadjuvant chemotherapy compared to those who have undergone primary surgery (OR = 19.26). Neoadjuvant chemotherapy was the strongest factor associated with positive resection margins in this study, *p* < 0.0001 (Figure 2a). Another statistically significant value we obtained was the association of the status of resection margins with the presence of DCIS. In our study, 11.2% of the patients in which lesions were highlighted and who had DCIS had positive resection margins (11 cases out of 98) compared with the patients who did not have such lesions, in which all the resection margins were negative. Thus, the presence of DCIS statistically significantly correlated with positive resection margins after breast-conservative surgery (*p* = 0.01) (Figure 2b).

The probability of obtaining at least one positive resection margin for young patients was higher (18.1%) compared with patients operated on after 40 years of age (6.8%). The age of the patient was not statistically significantly associated with the positive resection margins (*p* = 0.2).

The body mass index was not statistically associated with the positive resection margins.

The size of the tumor measured microscopically revealed an average of 15 mm. In two patients who underwent neoadjuvant chemotherapy with a complete pathological response, the tumor was not identified on the operative specimen. The smallest tumor measured 4 mm and the largest one was 35 mm. Considering the average size of the tumor, we set a cut-off of 15 mm. In 46 patients (3 patients with positive resection margins and 43 with negative resection margins), the microscopically measured tumor had a size smaller than 15 mm, and 59 patients had a tumor over 15 mm in size (5 patients with positive resection margins and 54 with negative resection margins). From a statistical point of view, positive resection margins were not associated with the tumor size (*p* = 0.75).

All the ultrasound-visible lesions were evaluated both preoperatively (when the drawing of the breast was made marking the tumor) and intraoperatively.

The patients who underwent neoadjuvant chemotherapy had a pre-therapy intratumoral marking clip.

Regarding the histological type of the invasive tumor, positive resection margins were identified only in the case of NST-type tumors in 9.4% of cases (11 cases out of 116).

The histological type and grade of invasive tumor were not statistically significantly associated with the positive resection margins (*p* = 0.3, respectively *p* = 0.6).

The molecular profile of the tumor was determined in 114 patients. None of the immunohistochemical profiles were statistically significantly associated with the positive resection margins (*p* = 0.31).

Multifocal tumors were identified in 10.4% of the total patients, of which 20% were associated with positive resection margins. For patients with unifocal tumors, the probability of identifying at least one positive resection margin in our study was 6.2%. Thus, *p* = 0.092, a value that tends to statistically associate the positive resection margins with multicentricity.

Microcalcifications, lymphovascular embolus, tumor necrosis, and inflammatory infiltrate were not statistically significantly associated with positive resection margins. However, in the case of microcalcifications, the odds ratio registered a value of 6770.

In total, 63 patients benefited from wide excision through an oncoplastic technique, and 80 patients did not undergo an oncoplastic procedure. Positive resection margins were identified in 8.5% of cases in the first category, respectively in 6.9% of classic wide excision cases. The technique of oncoplastic surgery was not statistically significantly associated with the positive resection margins (*p* = 1.00).

They were not statistically significantly associated with any of the axillary surgeries (*p* = 0.3).

We did not find a statistically significant association between the status of the axillary lymph nodes and the surgical resection margins. In 7.9% of the cases with negative axillary lymph nodes, at least one margin of surgical resection after the wide excision was positive compared with 7.1% for the patients whose lymph nodes were tumor invasive. Since we did not observe a statistical association between the status of the axillary lymph nodes and the positive resection margins, we did not correlate the survival and local recurrence with the axillary status.

In Table 1, all the studied parameters are represented (Table 1).

## 4. Discussion

The percentage of patients with positive resection margins after breast-conserving surgery was between 20% and 40% in most international studies [11,12,13], but we did not find data regarding the use of intraoperative ultrasound. In this study, in 11 patients, positive margins of resection were identified, which represents a percentage of 7.69%, and according to the recommendations of the guides, they were re-excised either by another conservative procedure or by mastectomy. This was associated with local recurrence compared to patients who had negative resection margins after the first intervention. Also, all the ultrasonographic detectable tumors underwent wide excision under ultrasound control for better appreciation of the surgical resection margins, including the patients with neoadjuvant chemotherapy and those with DCIS. In order to reduce the risk, we used intraoperative ultrasound and ultrasound and mammography of the surgical specimens.

Various methods have been developed to prevent post-chemotherapy positive resection margins. Marking the tumor with a clip pre-treatment and preoperative MRI are recommended for patients receiving neoadjuvant chemotherapy to evaluate the response to chemotherapy and guide surgery, especially in the case of Her2-positive tumors, where the risk of scattered response is higher. For DCIS, mammography of the operative specimen or bracketing (marking the edge with harpoons) could be useful. Also, a frozen section of the resection margins could be performed in patients at increased risk for positive resection margins.

At this time, there is no perfect method or system to appreciate intraoperative positive resection margins. The research in this field is still ongoing.

Several studies from the literature published between 2012 and 2016 support the fact that the tumor size, grade, location, IHC profile, axillary lymph node status, and body mass index of the patient are not associated with positive surgical resection margins in breast-conserving surgery [14]. The same results were obtained in our study.

The presence of DCIS and multicentric tumors are risk factors for positive resection margins according to several studies [11,12,14,15,16]. In our study, the presence of DCIS was associated with positive resection margins (*p* = 0.01), and in the case of multifocal tumors, the *p* value tended to be associated with positive resection margins (*p* = 0.09).

The presence of microcalcifications, lymphovascular tumor embolus, necrosis, or inflammatory infiltrate was not associated with positive resection margins. Contrary to our results, there are studies that show that microcalcifications are a predictive factor for positive margins [13].

Also, different results from other studies have been found regarding the histological type of the tumor. In our study, all 11 patients with positive resection margins had invasive ductal carcinoma out of a total of 116 who had this histological type. Invasive lobular carcinoma was detected in 5 patients, and other types were detected in 13 patients, but in these, no positive resection margin was identified. In the literature, there are several studies according to which the lobular type is a predictive factor for positive resection margins [11,15,16].

In a French study published in 2015 that evaluated positive resection margins after oncoplastic surgery of level II of the breast, the rate of positive resection margins was 11.9%, and the average size of the tumors was 26 mm; thus, the rate of positive margins was smaller despite the larger tumor size [14]. In our study, patients operated on using oncoplastic techniques had a mean tumor size of 14 mm, and the positive margin rate was 7.93% compared to patients operated on through classical wide excision, whose mean size was greater (21 mm), and the rate of positive margins was slightly lower (7.5%). We mention that through oncoplastic surgery techniques, we did not necessarily excise a larger amount of breast tissue, which is why these techniques were not associated with a low rate of positive resection margins.

According to the National Institute for Health and Clinical Excellence (NICE), preoperative chemotherapy may reduce tumor size to prevent mastectomy, but there is a slightly increased risk of local recurrence compared with radical surgery, which is why more studies in the literature have excluded patients who have benefited from neoadjuvant chemotherapy [14,15,16,17]. In our study, preoperative chemotherapy was administered to 34 patients, of which 9 had positive resection margins (26.47%) compared to 1.83% of positive margins for the patients who underwent primary surgery. Thus, neoadjuvant chemotherapy is a predictive factor for positive resection margins in breast-conserving surgery.

Regarding the postoperative follow-up of the patients, in our study, neither the positive resection margins nor the adjuvant radiotherapy influenced the patients’ survival or the local tumor recurrence. Several studies in the literature in recent years support the superiority of conservative surgery followed by radiotherapy compared with mastectomy in terms of patient survival and local recurrence of the disease [18,19,20,21,22,23,24].

In a follow-up period between 5 months and 20 years, the local recurrence varies between 2% and 14.5% depending on the time of evaluation, with differences related to the age of the patients and the histopathological and immunohistochemical characteristics of the malignancies. Also, according to the same studies, the survival is between 91% and 93% at 5 years, between 77% and 86% at 10 years, and 63.7% at 20 years [6,25,26].

In our study, we used ultrasound for the intraoperative localization of the tumor. According to a study by Parisi, S et al., LOCalizer™ appears to be a promising system for breast localization, especially in multiple lesions [27].

In our study, we included only patients who underwent neoadjuvant chemotherapy protocol treatment. According to Corrado Tinterri et al., the study The Impact of Different Patterns of Residual Disease on Long-Term Oncological Outcomes in Breast Cancer Patients Treated with Neo-Adjuvant Chemotherapy demonstrated that the interruption of NAC cycles and the size of the post-NAC tumor > 18 mm for any recurrence are factors that affect relapse and survival [28].

### The Limitations of the Study

According to the sample calculation formula, at a breast cancer incidence of 7895 cases per 100,000 women in Romania in 2013, the sample size should have been 112 patients with 5% accuracy, 174 patients with a precision of 4%, and 310 patients with a precision of 3%. In our study, 143 patients were evaluated.

$$n=\frac{Z_{\alpha }^{2}\cdot f(1-f)}{k^{2}}$$

*f*—frequency

*k*—precision of estimation

*Z_α_*—the value of z for a risk, *α* = 0.05

Another limitation of the study is represented by the 46 patients that were missed at follow-up.

Also, another limitation of the study is represented by the lack of information about the type of neoadjuvant chemotherapy received by the patients.

The retrospective nature of the analysis and the single-institution focus may introduce biases.

## 5. Conclusions

In conclusion, neoadjuvant chemotherapy and the presence of DCIS were statistically significantly associated with positive resection margins. These factors should be considered in the surgical management of early breast cancer.

## Figures and Tables

**Table 1:** All the studied parameters.

	Parameter		No. of Patients	Patients with PRM		No. of Patients with NRM	*p* Value	OR (95% CI)
				**nr.**	**%**			
1.	Patient’s age	<40	11	2	18.18	9	0.2017	3.037 (0.5686 to 16.22)
		>/=40	132	9	6.8	123		
2.	BMI	<19	3	0	0	3	0.5433	1.132 (0.05232 to 24.485)
		19–24	57	6	10.52	51		referent
		>/=25	83	5	6.02	78		1.835 (0.5319 to 6.333)
3.	Tumor diameter	<15 mm	61	4	6.55	57	0.7588	0.7519 (0.2099 to 2.694)
		>/=15	82	7	8.53	75		
4.	Histological type	NST	116	11	9.48	115	0.3946	referent
		lobular	5	0	0	5		1.199 (0.06219 to 23.118)
		others	13	0	0	13		2.943 (0.1639 to 52.865)
5.	Tumor grade	1	56	4	7.14	52	0.6187	referent
		2	61	6	9.8	55		0.7051 (0.1882 to 2.642)
		3	26	1	3.8	25		1.923 (0.2041 to 18.122)
6.	Molecular profile	A	44	1	2.27	43	0.3129	referent
		B Her 2+	4	1	25	3		0.06977 (0.003441 to 1.415)
		B Her2-	49	4	8.16	45		0.2615 (0.02809 to 2.436)
		Her 2	2	0	0	2		0.1724 (0.005496 to 5.409)
		TN	6	0	0	6		0.4483 (0.01644 to 12.225)
7.	DCIS	yes	98	11	11.22	87	0.0173	11.96 (0.6886 to 207.7)
		no	45	0	0	45		
8.	Multicentricity	U	128	8	6.25	120	0.0923	0.2667 (0.06231 to 1.141)
		M	15	3	20	12		
9.	Microcalcifications	yes	63	7	11.11	56	0.1845	6.770 (0.3720 to 123.2)
		No	25	0	0	25		
10.	Lymphovascular embolus	yes	55	6	10.9	49	0.2993	2.490 (0.5921 to 10.47)
		no	64	3	4.6	61		
11.	Tumor necrosis	yes	41	5	12.19	36	0.1428	3.102 (0.7005 to 13.73)
		no	70	3	4.28	67		
12.	Inflammatory infiltrate	yes	108	7	6.48	101	0.4838	0.5545 (0.06046 to 5.085)
		no	9	1	11.11	8		
13.	Axillary procedure	ALND	38	3	7.89	35	1.0000	1.039 (0.2608 to 4.141)
		SLNB	105	8	7.61	97		
14.	Axillary lymph nodes	N0	101	8	7.92	94	1.0000	1.106 (0.2787 to 4.392)
		N1	42	3	7.14	39		
15.	Oncoplastic procedure	yes	63	5	7.93	58	1.0000	1.063 (0.3089 to 3.659)
		no	80	6	7.5	74		
16.	Neoadjuvant chemotherapy	yes	34	9	26.47	25	<0.0001	19.26 (3.915 to 94.75)
		no	109	2	1.83	107		

PRM–positive resection margins; NRM–negative resection margins; BMI–body mass index; OR–odds ratio; CI–confidence interval; DCIS–ductal carcinoma in situ; A–Luminal A; BHer2+–Luminal B Her2 positive; B Her2- –Luminal B Her 2 negative; TN–triple negative; U–unifocal tumors; M–multifocal tumors; ALND–axillary lymph nodes dissection; SLNB–sentinel lymph node biopsy.

## Data Availability

Data are contained within the article.
